# *MMWR* Express App for iPhone and iPad Now Available

**Published:** 2014-06-27

A new *MMWR* application, *MMWR* Express, is now available for free download in the Apple App Store for both iPhone and iPad. This application provides fast access to the blue summary boxes in the *MMWR Weekly*. Summaries can be viewed by publication date or by searching for a specific subject (e.g., *Salmonella*). It is the first iPhone/iPad app to provide *MMWR* content.

*MMWR* publications have been in existence since 1952, and today *MMWR* remains CDC’s primary vehicle for scientific publication of timely, reliable, authoritative, accurate, objective, and useful public health information and recommendations. *MMWR* readership, which extends around the globe, predominantly consists of physicians, nurses, public health practitioners, epidemiologists and other scientists, researchers, educators, pharmacists, and laboratorians.

This application is one of an expanding collection of mobile applications from CDC. Development of applications for other mobile operating systems is under consideration. When online, *MMWR* Express can quickly check for new content, ensuring that users always have the most up-to-date information. Users also can share content with others via e-mail, text message, Facebook, or Twitter. The free application is available at https://itunes.apple.com/us/app/mmwr-express/id868245971?mt=8.

